# PhosphoTyrosyl Phosphatase Activator of *Plasmodium falciparum*: Identification of Its Residues Involved in Binding to and Activation of PP2A

**DOI:** 10.3390/ijms15022431

**Published:** 2014-02-11

**Authors:** Audrey Vandomme, Aline Fréville, Katia Cailliau, Hadidjatou Kalamou, Jean-François Bodart, Jamal Khalife, Christine Pierrot

**Affiliations:** 1 Center for Infection and Immunity of Lille, Inserm U1019-CNRS UMR 8204, University of Lille Nord de France, Institut Pasteur de Lille, 1 Rue du Professeur Calmette, Lille 59019, Cedex, France; E-Mails: audrey.vandomme@pasteur-lille.fr (A.V.); alinekatia.freville@gmail.com (A.F.); hkalamou@gmail.com (H.K.); 2 EA4479, IFR147, Laboratoire de Régulation des Signaux de Division, SN3, Université des Sciences et Technologies de Lille, Villeneuve d’Ascq 59655, France; E-Mails: katia.maggio@univ-lille1.fr (K.C.); Jean-Francois.Bodart@univ-lille1.fr (J.-F.B.)

**Keywords:** *Plasmodium*, PTPA, PP2A, phosphatase, dephosphorylation

## Abstract

In *Plasmodium falciparum* (Pf), the causative agent of the deadliest form of malaria, a tight regulation of phosphatase activity is crucial for the development of the parasite. In this study, we have identified and characterized PfPTPA homologous to PhosphoTyrosyl Phosphatase Activator, an activator of protein phosphatase 2A which is a major phosphatase involved in many biological processes in eukaryotic cells. The PfPTPA sequence analysis revealed that five out of six amino acids involved in interaction with PP2A in human are conserved in *P. falciparum*. Localization studies showed that PfPTPA and PfPP2A are present in the same compartment of blood stage parasites, suggesting a possible interaction of both proteins. *In vitro* binding and functional studies revealed that PfPTPA binds to and activates PP2A. Mutation studies showed that three residues (V^283^, G^292^ and M^296^) of PfPTPA are indispensable for the interaction and that the G^292^ residue is essential for its activity. In *P. falciparum*, genetic studies suggested the essentiality of PfPTPA for the completion of intraerythrocytic parasite lifecycle. Using *Xenopus* oocytes, we showed that PfPTPA blocked the G2/M transition. Taken together, our data suggest that PfPTPA could play a role in the regulation of the *P. falciparum* cell cycle through its PfPP2A regulatory activity.

## 1. Introduction

Most processes in eukaryotic cells are under the control of protein-protein interactions, notably in the case of enzymes which are regulated not only by post translational modifications but also by protein interacting partners that direct and control their activities. Protein phosphatases are a group of enzymes that counterbalance the action of kinases to modulate proteins functions via the modifications of their phosphorylation status. Unlike kinases, phosphatases belong to several distinct protein/gene families and their number, putatively or experimentally identified in different organisms, is still fewer than kinases [1,2]. This difference, together with the fact that phosphatases dephosphorylate diverse substrates *in vitro*, has often raised the question about the specificity of phosphatases and their regulation. Based on the observations accumulated in the past decade, mainly on the two major serine/threonine phosphatases PP1 and PP2A, there is now clear-cut evidence that the localization, substrate specificity and magnitude of activity is accomplished by the capacity of the catalytic subunit to interact with diverse regulatory proteins. For PP1, more than 200 interacting proteins have been identified using a variety of biochemical, functional and genetic approaches among which 129 proteins have been characterized [3–5]. In contrast, there are only seven regulators described for PP2A [6–11] including I2^PP2A^ and PhosphoTyrosyl Phosphatase Activator (PTPA) which are the best characterized regulators. For both phosphatases, it has been observed that several regulators are as essential as the catalytic subunit itself. In the case of PP2A, converging studies have demonstrated the involvement of PTPA, known also as PP2A activator protein, in the activation loop of PP2A and in cell growth and survival [12–16]. PTPA was first purified from rabbit skeletal muscle and *Xenopus laevis* oocytes, for its specific capacity to activate PP2A *in vitro* [6]. This activation has been shown to be ATP- and Mg^2+^-dependent [6]. Subsequently PTPA was cloned from several species and sequence alignment revealed a high degree of identity (>85%) [17,18]. More recently, PTPA was described to exhibit a peptidyl prolyl isomerase activity (PPIase) [19] and the target seems to be the proline 190 (P^190^) of PP2A which is conserved in all PP2A. This novel activity seems to be associated with the ability of PTPA to activate PP2A as it requires the presence of ATP-Mg^2+^ and the mutant PTPA with abolished PP2A activating function did not show any PPIase activity. In addition, the mutated version of PP2A (P^190^A) has been shown to be partially active but became insensitive to the activation by PTPA [19], indicating that this proline is essential for the activation process to take place.

The crystal structures of human PTPA (HuPTPA) and yeast orthologs have been determined [20–22]. These structures showed that PTPA has an overall α-helical structure and identified a highly conserved cleft as a potential region for interaction. On the basis of HuPTPA structure, the interaction between PP2A and different PTPA mutants investigated by GST-pull down revealed that six residues out of 18 (V^209^, E^270^, V^281^, G^290^, M^294^ and K^302^) strongly contributed to the binding to PP2A [20]. Although the diverse biochemical functions of PTPA on PP2A detected *in vitro* remain to be better defined under the global cellular spatiotemporal constraints, *in vivo* studies revealed that its disruption is lethal in yeast by controlling the progression of the G1 phase of the cell cycle [15] and the G2/M transition [13].

In *Plasmodium falciparum*, the most virulent and deadly parasite for humans, a number of phosphatases have been characterized, including PP1 and PP2A [23–39]. Despite the importance of these enzymes and their direct implication in cell cycle shown in many eukaryotic cells other than *Plasmodium*, very few experimental approaches have been carried out to identify their regulators. Recently, we have characterized three regulators of PfPP1, named PfLRR1 [40], Inhibitor 2 (PfI2) [41] and Inhibitor 3 (PfI3) [42]. PfLRR1 and PfI2, as expected, showed a strong capacity to inhibit the PfPP1 activity although they exhibit substantial differences in the binding motifs to PP1. Unexpectedly, PfI3, unlike its human counterpart clearly revealed an activation of PP1 and a lack to complement Inhibitor 3 deficient yeast [42]. Additionally, reverse genetic studies suggested that the above regulators seem to be essential for blood parasite growth. Finally, peptides derived from PfI2 and PfI3 competing with the main binding site to PP1 exhibited an anti-plasmodial activity against blood stage parasites *in vitro* [41,42].

With respect to PP2A, biochemical and chromatography approaches combined with a micro-sequencing procedure suggested the presence of a PP2A-like catalytic subunit in this parasite [26]. Subsequently, the availability of the *P. falciparum* genome, together with transcriptomic and proteomic studies confirmed its expression by different parasite stages. The first regulator of PfPP2A has been characterized by Dobson *et al*. [43]. These authors reported the expression by *P. falciparum* of a homolog to Inhibitor 2 of PP2A (PfI2^PP2A^), based on the fact that the primary structure of PfI2^PP2A^ exhibited an overall identity of 28% and 55% similarity with its human counterpart, that the PfI2^PP2A^ specifically inhibited PP2A and that its overexpression in HeLa cells led to an increase of phosphorylation of transcription factors. This protein is essential during the asexual erythrocytic cycle of *Plasmodium berghei* (rodent *Plasmodium* specie). Indeed, the Leiden Malaria Research Group and the Istituto Superiore di Sanita failed to disrupt the PbI2^PP2A^ gene by double crossing over (http://www.pberghei.eu/index.php?rmgm=246) despite the accessibility of its locus by PbI2^PP2A^-GFP tagging integration (http://www.pberghei.eu/index.php?rmgm=218). Although the exact physiological function of PfI2^PP2A^ is still to be examined, its location in the cytoplasm of *P. falciparum* strongly suggests that its regulatory function could take place in this compartment. No other regulators of PfPP2A have been characterized so far.

In the present study, we report the characterization of PfPTPA, the *P. falciparum* homolog of the human PhosphoTyrosyl Phosphatase Activator and show that this protein is able to directly bind to PP2A. *In vitro* binding studies with recombinant wild type or mutated proteins showed that three residues (V^283^, G^292^ and M^296^) of PfPTPA are important for this interaction. Functional studies revealed that PfPTPA activates PP2A *in vitro* and that five residues (E^272^, V^283^, G^292^, M^296^ and K^304^) are involved in a dramatic loss of function for the mutated PfPTPA G^292^A protein. In *P. falciparum*, genetic studies suggested the essentiality of PfPTPA for the completion of the intraerythrocytic parasite life cycle. Further studies, using a heterologous model, showed that PfPTPA interacted with endogenous PP2A and that the microinjection of PfPTPA to *Xenopus* oocytes blocked the G2/M transition.

## 2. Results and Discussion

### 2.1. Molecular Cloning and Sequence Analysis of PfPTPA

**Figure 1.**
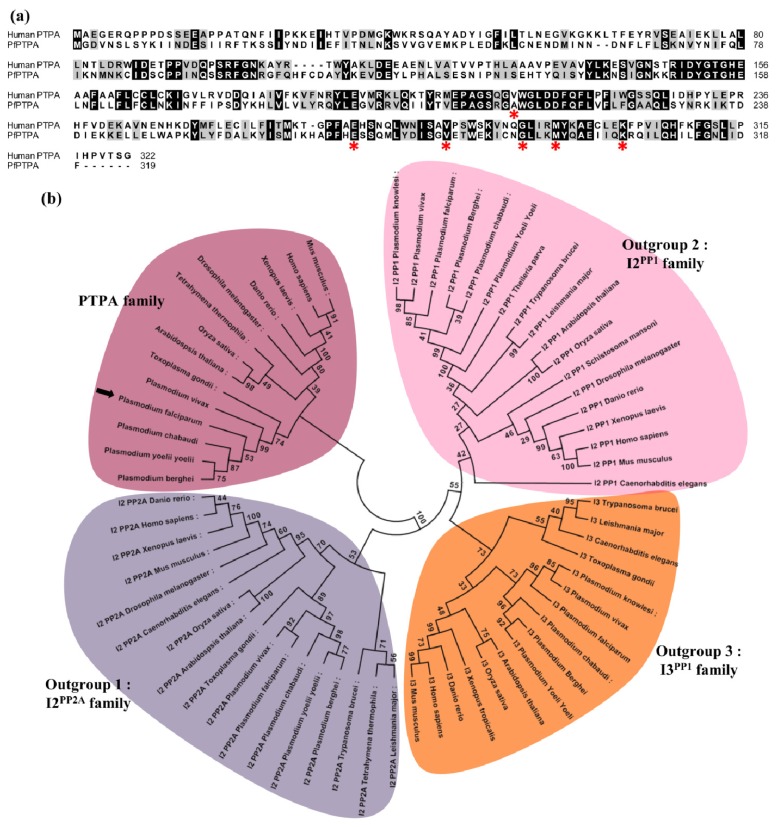

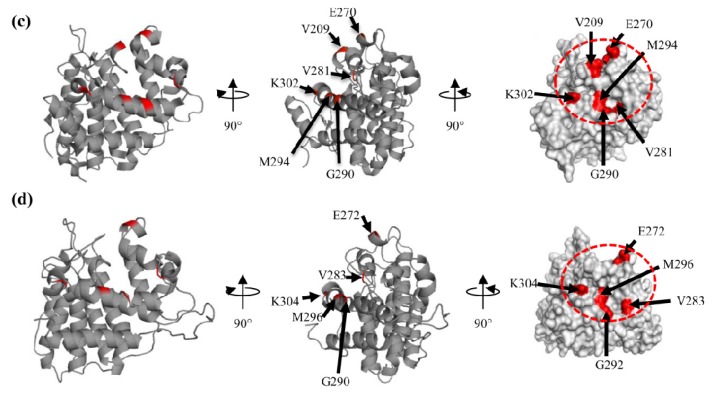
Molecular cloning and sequence analysis of PfPTPA. (**a**) Analysis of PfPTPA (PF3D7_1430100) amino acid sequence. PfPTPA was aligned with the human PTPA (CAA60163.1) using ClustalW Multiple Alignment (BioEdit). The identical residues are highlighted in black and similar residues in grey. Stars symbolized amino acids involved in PTPA/PP2A interaction in human; (**b**) Phylogenic tree of the PTPA family. A maximum likelihood tree was generated from the 21 PTPA sequences using MEGA5 [45] under the JTT + G + I model with 100 bootstrap repetitions. Outgroups are formed by PP2A inhibitor 2 orthologs (I2^PP2A^, outgroup 1) PP1 inhibitor 2 orthologs (I2^PP1^, outgroup 2) and PP1 inhibitor 3 orthologs (I3^PP1^, outgroup 3); (**c**) Crystal structure of HuPTPA (PDB: 2IXM). Amino acid residues involved in PTPA/PP2A interaction are shown in red; (**d**) Structural model of PfPTPA based on the crystal structure of HuPTPA (PDB: 2IXM) using the ModBase server [46]. Amino acid residues studied in this work for the PTPA/PP2A interaction are shown in red.

The *P. falciparum* genome encodes a single putative PTPA (PF3D7_1430100, 319 amino acids, 37.4 kDa). The predicted gene contained 5 exons but had not yet been cloned and identified experimentally. We isolated a RT-PCR product from *P. falciparum* blood stage total RNA using primers spanning the full length sequence. The complete sequencing of five independent clones showed an open reading frame of 960 bp (Supplementary Figure S1), confirming the ORF annotated in PlasmoDB [44] (www.plasmodb.org). Reciprocal BLASTP analysis using the deduced amino acid sequence as query confirmed the homology of this gene product with other known PTPA. The comparison with its human counterpart revealed an overall identity of 32% (54% homology), reaching a maximum of 40% in the C-terminal region of the sequence (Figure 1a). PfPTPA orthologs are also present in all sequenced rodent and human *Plasmodium* species. The ClustalW program was then used to generate an alignment of the *Plasmodium* PTPA sequences with those of other species including human and *Toxoplasma gondii* sequences. As outgroups, inhibitors of PP1 and PP2A were included. The resulting alignment was used to generate a maximum likelihood phylogenetic tree. The data presented in Figure 1b clearly shows that *Plasmodium* PTPAs form a divergent subgroup along with *T. gondii* but cluster in the group of other known PTPAs, confirming the relatedness of PfPTPA to PhosphoTyrosyl Phosphatase Activator family (Figure 1b).

For the human PTPA, it should be noted that the analysis of the crystal structure combined with amino acid mutation studies revealed that six amino acids present in the C-terminal region of the protein (V^209^, E^270^, V^281^, G^290^, M^294^ and K^302^) are critical for its function on PP2A [20]. In *P. falciparum* five amino acids out of six were found to be conserved in PfPTPA and correspond to E^272^, V^283^, G^292^, M^296^ and K^304^ positions (Figure 1a). To shed further light on the position of these conserved residues in the global structure of PfPTPA, a 3D model was built using human PTPA (PDB: 2IXM) as a template (https://modbase.compbio.ucsf.edu/scgi/modweb.cgi) (Figure 1c,d).

The overall structure showed some differences between human and *P. falciparum* homologs. Indeed, human PTPA is composed of thirteen α helices and four β sheets while PfPTPA is composed of sixteen α helices but no β sheet. The α helices are localized almost identically between both proteins however four helices in HuPTPA are subdivided into eight shorter helices in PfPTPA (Supplemental Figure S2). This model shows that residues involved in PP2A interaction in human are on identical structures except residues G^292^ and K^304^ which are in an α helix structure in human but not in *P. falciparum*. Despite these differences, both the crystal of human PTPA and the PfPTPA model showed that all residues are at the periphery as shown in Figure 1c,d. This similarity suggests that some of the residues involved in the binding domain to PP2A could be conserved in human and *P. falciparum* PTPA.

### 2.2. Expression of PfPTPA and PfPP2A by *Plasmodium falciparum* and Localization

**Figure 2.**
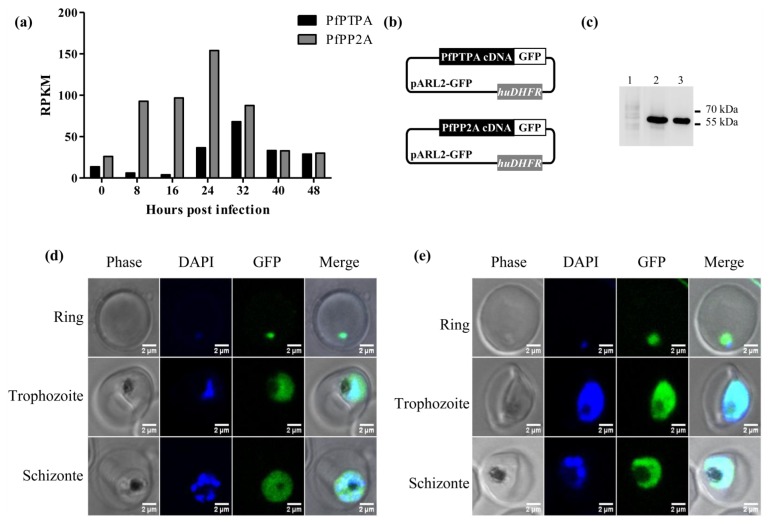
Expression of *pfptpa* and *pfpp2a* gene by *Plasmodium falciparum* and localization (**a**) Representation of *pfptpa* (black bars) and *pfpp2a* (grey bars) RNA expression during the erythrocytic life cycle of *P. falciparum*. Data plotted are from PlasmoDB [44]; (**b**) Schematic representation of the pARL2-*pfptpa-gfp* and pARL2-*pfpp2a-gfp* vectors used for episomal expression of both PfPTPA and PfPP2A; (**c**) The expression of both proteins was checked by western blotting with anti-GFP antibodies after separation on 15% SDS-PAGE. Lane 1 represents the extract of wild type parasites. Lane 2 and 3 represent extracts from PfPTPA and PfPP2A transfected parasites respectively. Expression and localization of PfPTPA-GFP (**d**) and PfPP2A-GFP (**e**) throughout the erythrocytic cell cycle of *P. falciparum* were analyzed by fluorescence microscopy after transfection as described under experimental section.

RNA-Seq data available at the PlasmoDB [44] showed that the gene was transcribed in blood stage parasites, with a peak in late trophozoites (32 h). PfPTPA transcripts were also identified in gametocytes and the protein was detected via proteomics in all asexual and sexual parasites examined [47–49]. Because PTPA is an activator of PP2A [6,20,50], we plotted the RNA-Seq data of the latter along with those obtained for PfPTPA. Results depicted in Figure 2a showed that both transcripts were detectable at early stage trophozoites with a higher level for PP2A transcripts (24 h post infection). In late trophozoites/schizonts (32–40 h post infection), the relative abundance of both transcripts was similar, suggesting that PfPP2A could be activated through PfPTPA during the late developmental stages of the intraerythrocytic lifecycle.

Based on the above observations, we sought to follow up the distribution of PfPTPA and PfPP2A during the intraerythrocytic development cycle. To this end, the *P. falciparum* 3D7 strain was transfected with pARL2 constructs mediating the episomal expression of *pfptpa* or *pfpp2a-gfp* full-length sequences (Figure 2b). The use of this vector by Kuhn *et al*. showed that the trafficking was dependent on the fused protein rather than on the *pfcrt* promoter used [51]. Using a monoclonal anti-GFP antibody, immunoblot analysis of a total extract of blood stage parasites expressing either PfPTPA-GFP or PP2A-GFP revealed the presence of specific bands at the expected size of each fused protein (Figure 2c, lanes 2 and 3), demonstrating the integrity of the fused protein in transfected parasites.

Examination of live parasites transfected with *pfptpa-gfp* showed that the signal was confined within the parasite where the distribution is nucleo-cytoplasmic in rings, trophozoites and schizonts (Figure 2d), as the fluorescence partially overlapped DNA staining. Parasites transfected with the construct containing *pfpp2a-gfp* showed a nucleo-cytoplasmic distribution in rings and schizonts with an intense accumulation in the nucleus of late trophozoites (Figure 2e). These results are in accordance with previous localization studies in mammalian cells showing that PTPA and PP2A have a nucleo-cytoplasmic localization with an accumulation in the nucleus when human cells progressed into S phase [52], and in the Fission Yeast [12] respectively. The PfPTPA-GFP or PfPP2A-GFP signal was completely absent from the digestive food vacuole and the cytoplasm of red blood cells (Figure 2d,e). The presence of PfPP2A and PfPTPA in both cytoplasm and nuclear compartments of the parasite suggests a potential interaction between these proteins and raises the question of a potential regulatory effect on PP2A activity.

### 2.3. Binding of PfPTPA to PP2A

**Figure 3.**
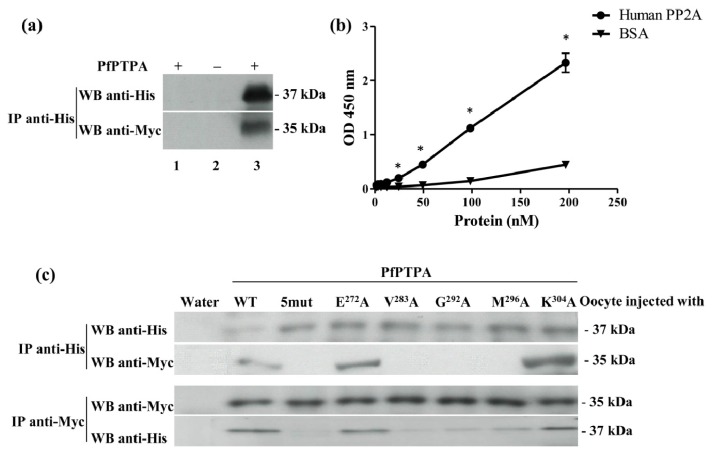
Interaction studies of PfPTPA to PP2A. (**a**) Binding of PfPTPA with PfPP2A in *Xenopus* oocyte. Co-immunoprecipitation of the PfPTPA-PfPP2A complex with anti-His antibodies (recognizing recombinant PfPTPA tagged with 6-His) (lanes 2 and 3) from microinjected *Xenopus* extracts. The anti-mouse IgG antibody (lane 1) was used as a control. Immunoprecipitates from *Xenopus* oocytes microinjected with water (−) or PfPTPA (+) were eluted and separated by SDS-PAGE and transferred to nitrocellulose membrane. Immunoblot analysis was performed with anti-His antibodies (recognizing PfPTPA) (**upper panel**) or anti-Myc antibodies (recognizing PfPP2A) (**lower panel**); (**b**) Interaction of PfPTPA with HuPP2A *in vitro* assessed by ELISA based assay. Increasing quantities of biotinylated recombinant PfPTPA were added to wells coated with human PP2A (100 ng/well) or BSA (100 ng/well) as a negative control. Results are means ± SEM of two independent experiments performed in duplicate (stars (*) represent significant differences *p* = 0.01); (**c**) Binding of mutated PfPTPA with PfPP2A in *Xenopus* oocytes. Co-immunoprecipitation experiment of the PfPTPA-PfPP2A complexes with anti-His antibodies (recognizing recombinant wild type and mutated PfPTPA) (**upper panel**) or with anti-Myc antibodies (recognizing PfPP2A) (**lower panel**) from microinjected *Xenopus* oocytes. Immunoprecipitates from *Xenopus* oocytes microinjected with water, WT or mutated PfPTPA were eluted and separated by SDS-PAGE and transferred to nitrocellulose membrane. Immunoblot analysis was performed with anti-His or anti-Myc antibodies.

Next, the interaction of PfPTPA and PfPP2A was investigated. The production of these proteins in *E. coli* revealed that PfPTPA could be produced soluble under non-denaturing conditions, while PfPP2A could not be obtained under its active form as it did not show any activity against pNPP, a canonical substrate for phosphatases. In order to overcome this point, we used *Xenopus* oocytes which we previously reported to be able to produce *Plasmodium* proteins [40]. Hence, we injected oocytes with the recombinant His tagged-PfPTPA protein and the cRNA corresponding to Myc tagged-PfPP2A protein. Oocyte lysates were prepared as mentioned in the Experimental section and used for co-immunoprecipitation/western blot experiments. As shown in Figure 3a, immunoblot analysis of PfPTPA immunoprecipitates using anti-tag antibodies showed that PfPP2A had been co-immunoprecipitated with PfPTPA. To further ascertain a direct interaction of PfPTPA with PP2A, together with the fact that PP2A is highly conserved among different species, a human PP2A commercially available was used for binding in an ELISA based assay. Results presented in Figure 3b evidenced the capacity of PfPTPA to bind to coated HuPP2A and the intensity of the signal was dependent on the amount of PfPP2A added. Taken together, our data demonstrate that PfPTPA physically interacts with PfPP2A and support previous results showing a direct interaction between PTPA and PP2A [53].

The fact that PTPA is conserved across species suggests that it may use a conserved set of amino acids to interact with PP2A. Based on the sequence comparison depicted in Figure 1a, five out of six residues involved in human PTPA/PP2A interaction are conserved in *Plasmodium*. To further explore the role of conserved residues of PfPTPA in binding to PfPP2A, five PfPTPA versions containing a single mutation of a critical amino acid residue were produced as recombinant proteins and used in co-immunoprecipitation assays. As control, a PfPTPA protein containing the five mutations (PfPTPA 5mut) was also produced and tested under the same conditions. Co-immunoprecipitation assays were performed on lysates from oocytes injected with wild or mutant His tagged-PfPTPA proteins (PfPTPA E^272^A, PfPTPA V^283^A, PfPTPA G^292^A, PfPTPA M^296^A and PfPTPA K^304^A) along with the cRNA translating the Myc tagged-PfPP2A. Results showed that the binding of mutants E^272^A and K^304^A was not impaired while the binding of mutants V^283^A, G^292^A and M^296^ was abolished (Figure 3c). These results suggest a differential binding domain in human and *Plasmodium*. Indeed, in HuPTPA the E^270^ residue is essential for the interaction as well as residues G^290^ and M^294^ while the residues V^281^ and K^302^ are less important. Finally, the major difference between human and *Plasmodium* binding of PTPA to PP2A concerns the E^270^ residue (corresponding to E^272^ in *P. falciparum*) which is critical in human but not in *Plasmodium*. This difference suggests a differential binding domain that may be related to a difference in the tertiary structure of PTPA in both organisms. It is notable that the version of PfPTPA protein containing all five mutations did not interact with PfPP2A. Taken together, our data confirm that V^283^, G^292^ and M^296^ residues of PfPTPA are involved in interaction with PfPP2A.

### 2.4. Effect of PfPTPA on the Activity of PP2A

**Figure 4.**
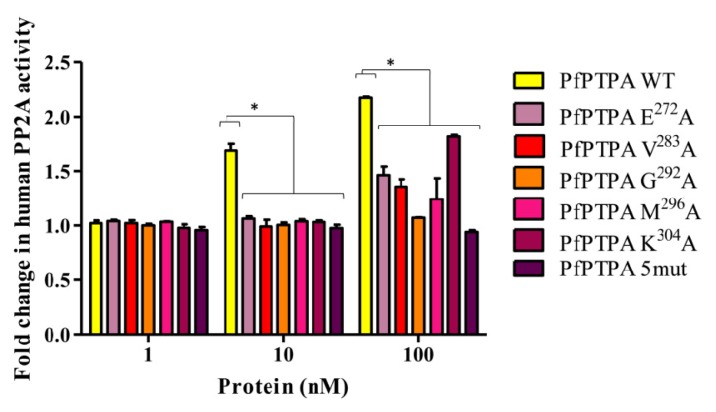
Effect of wild type and mutated PfPTPA proteins on PP2A activity. The capacity of PfPTPA (WT or mutated) to regulate HuPP2A was assessed using pNPP activity tests. The human PP2A activity was measured at 405 nm by the release of p-nitrophenol after incubation with different concentration of recombinant WT or mutated PfPTPA proteins. Results presented as fold change in human PP2A activity are means ± SEM of three independent experiments (stars (*) represent significativity *p* = 0.01).

It has been previously reported that PTPA proteins are able to regulate PP2A by increasing its activity towards different substrates including, but not limited to, pNPP substrate [6]. Because all assays to produce recombinant active PfPP2A were unsuccessful, and as PfPTPA is able to bind HuPP2A (Figure 3b), the function of PfPTPA on the activity of HuPP2A was assessed. Using a concentration of human PP2A within a range producing linear release of phosphate, the effect of wild-type and mutated recombinant PfPTPA proteins was evaluated as described in the Experimental section. Results showed a significant increase in the phosphatase activity (up to 200%) when the PP2A was preincubated with PfPTPA wild type (Figure 4). When PfPTPA mutated proteins were tested at 10 nM, we observed that all mutations led to an almost complete loss of function of PfPTPA (Figure 4). At higher contentration (100 nM), only the PfPTPA G^292^A protein was still unable to activate PP2A, however the other four mutated proteins showed a slight increase of PP2A activity. The PfPTPA protein containing the five mutations was inactive at any concentration used. These data, together with the co-immunoprecipitation experiments suggest that G^292^ is a vital and a primary residue for the activity of PfPTPA. It is important to remember that in human PTPA all residues except K^302^ are equally essential for its activity [20]. The fact that PfPTPA exhibits only five residues out of six, together with the key role of G^292^, suggest a particular interaction between PfPTPA and PfPP2A to adequately adapt the activation of this enzyme towards specific substrate in *Plasmodium*.

### 2.5. Genetic Manipulations of *pfptpa* and *pfpp2a* in *Plasmodium falciparum*

**Figure 5.**
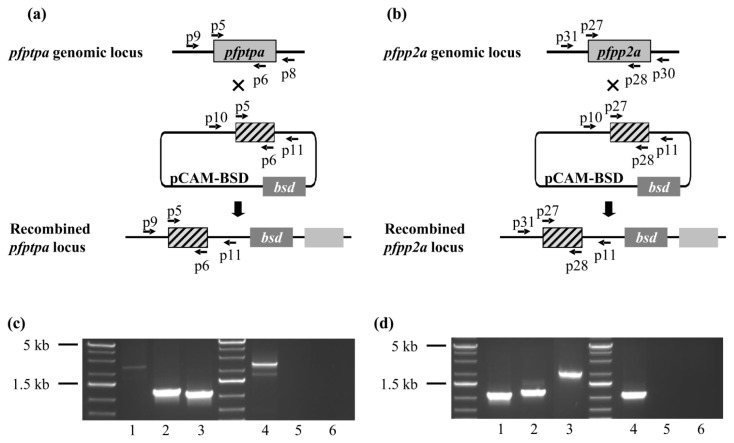
Genetic studies of *pfptpa* and *pfpp2a*. (**a**,**b**) Gene-targeting construct for gene disruption by single homologous recombination using the pCAM-BSD, and the locus resulting from integration of the knockout *pfptpa* (**a**) or *pfpp2a* (**b**) construct. (**c**,**d**) Analysis of pCAM-BSD-*pfptpa* (**c**) and pCAM-BSD-*pfpp2a* (**d**) transfected 3D7 culture by PCR; lanes 1–3 correspond to DNA extracted from transfected parasites; lanes 4–6 correspond to DNA extracted from wild type parasites. Lanes 1 and 4 represent the detection of the full length wild type locus (PCR with p9-p8 and p31-p30 respectively); lanes 2 and 5 represent the detection of episomal DNA (PCR with p10 and p11); and lanes 3 and 6 represent the detection of integration of the insert (PCR with p9-p11 and p31-p11 respectively).

It has been previously shown that the disruption of *pp2a* or *ptpa* ortholog genes in yeast results in lethality [14,54]. In mice, it was also reported that the homozygous null for PP2A detected at the embryonic stage were not viable [55]. To study whether the lack of PfPTPA and PfPP2A expression could affect the *Plasmodium* blood stage life cycle, we attempted to disrupt these genes by single crossing-over homologous recombination using the pCAM vector system (Figure 5a,b). We transfected blood ring stage parasites of the 3D7 strain with a pCAM-BSD-*pfptpa* or pCAM-BSD-*pfpp2a* constructs containing 5′ fragments derived from the respective genomic sequences and the *bsd* gene conferring resistance to blasticidin (Figure 5a,b). After transfections and drug treatments, the presence of each construct was checked by a plasmid rescue approach as previously described (data not shown). The integration in viable parasites was then analyzed by PCR on genomic DNA, using oligonucleotides presented in Supplemental Table S1. Template DNA from wild parasites was used as control. The amplicons corresponding to the wild locus as well as to the amplicons diagnostic for PfPTPA and PfPP2A were detectable in transfected parasites (Figure 5c lanes 1 and 3, Figure 5d lanes 1 and 3). The generation of mutant parasites demonstrates the accessibility of the locus. In order to further examine the phenotype of mutant parasites, we attempted to establish stable clonal lines by limiting dilution. Unexpectedly, we were unable to obtain viable clones which did not express either *pfptpa* or *pfpp2a* genes. This was confirmed by the fact that cloned parasites expressed PfPTPA or PfPP2A as detected by RT-PCR (not shown). This suggests that the initial genomic integration was transitory and/or that both genes are in fact essential at some stage for the completion of the intraerythrocytic cycle. The results related to PfPP2A are supported by previous studies reporting that okadaic acid, an inhibitor of phosphatase activities, was able to drastically block blood parasite growth *in vitro*, suggesting the essentiality of phosphatases including PfPP2A [56]. Further studies are still required to examine the essential functions and the precise timing of when and where these proteins are critical for the parasite growth/development. This should await the development of a powerful and robust inducible expression system for *Plasmodium falciparum* proteins. A complementary approach to study the function of these genes could be the use of the inducible system in rodent *Plasmodium* or *T. gondii*.

### 2.6. Inhibition of G2/M Transition of *Xenopus* oocytes by PfPTPA

**Figure 6.**
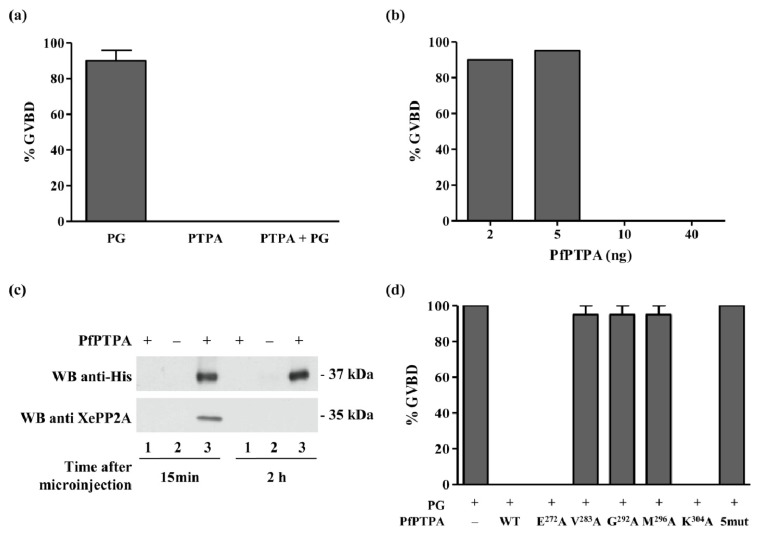
Inhibition of G2/M transition of *Xenopus* oocytes by PfPTPA. (**a**) Percentage of GVBD induced by the progesterone (PG), PfPTPA or the microinjection of PfPTPA followed by PG incubation; (**b**) Percentage of GVBD induced by PG incubation after microinjection of different amount of PfPTPA; (**c**) Binding of PfPTPA with XePP2A in *Xenopus* oocytes. Co-immunoprecipitation experiment of the PfPTPA-XePP2A complex with anti-His antibodies (recognizing recombinant PfPTPA tagged with 6-His) (lanes 2 and 3) from microinjected *Xenopus* oocytes. The anti-mouse IgG antibody (lanes 1) was used as a control. Immunoprecipitates from *Xenopus* oocytes microinjected with water (−) or PfPTPA (+) were eluted, separated by SDS-PAGE and transferred to nitrocellulose membrane. Immunoblot analysis was performed with anti-His antibodies (recognizing PfPTPA) (upper panel) or anti-XePP2A antibodies (lower panel); (**d**) Effect of PfPTPA on progesterone-dependent GVBD in *Xenopus* oocytes. Percentage of GVBD induced by PG after the microinjection of 10 ng of PfPTPA WT or mutated (*n* = 2).

PP2A is one of the major Ser/Thr phosphatases which dephosphorylate diverse proteins involved in the control of eukaryotic cell cycle [57–59]. In the context of *Xenopus* oocytes, inhibition of PP2A promotes G2/M transition by enabling the activation of the M-Phase Promoting Factor, universal factor of M-Phase entry. Thus, activity of PP2A contributes to block of oocytes in the G2-like state while antagonizing MPF activation [60]. G2/M transition in Xenopus oocytes can be triggered *in vitro* by hormonal stimulation through addition of progesterone in the medium [61], provoking an oocyte Germinal Vesicle Breakdown or GVBD. If PfPTPA activates PP2A, its preinjection in oocytes will lead to an inhibition of progesterone-induced maturation. With this rationale in mind, we assessed the impact of PfPTPA on the G2/M transition induced by progesterone. First, it is important to emphasize that the microinjection of PfPTPA alone did not produce any GVBD (Figure 6a), however, as expected the progesterone induced GVBD (Figure 6a). Results presented in Figure 6a showed that PfPTPA was able to block the GVBD induced by progesterone. The threshold of this inhibition started to be observed at 10 ng/oocyte (Figure 6b). In parallel, it was important to verify whether PfPTPA can bind *Xenopus* PP2A (XePP2A). As shown in Figure 6c, the use of specific PP2A antibodies for immunoblot analysis of eluates co-immunoprecipitated with anti-His antibodies revealed the presence of XePP2A in the complex as early as 15 mn post injection. This complex was not detectable 2 and 18 h post-injection, although PfPTPA was still present in oocyte extracts, suggesting an early detection and rapid action of PfPTPA. In this model, we propose that PfPTPA would activate PP2A which consequently dephosphorylates a key substrate required for the signal pathway activated by progesterone.

Next, we used this approach to evaluate the contribution to this activity of the amino acid residues involved in PP2A binding. Results showed that the mutations V^283^A, G^292^A and M^296^A completely abrogated the function of PfPTPA as GVBD was still observed after progesterone treatment (Figure 6d). It is notable that co-immunoprecipitation experiments showed that these three mutated proteins exhibited a strong decrease of their binding capacity to XePP2A (Supplemental Figure S4). Concerning E^272^A and K^304^A mutated proteins, no loss of function was detectable. Altogether, these data confirmed the role of the residue G^292^ and showed that the residues V^283^ and M^296^ participate also in the function of PfPTPA. However, the residues E^272^ and K^304^ did not seem to affect either the binding or the function of PfPTPA.

## 3. Experimental Section

### 3.1. Materials

Plasmid pETDuet-1 was purchased from Novagen. Plasmids pCAM-BSD and pCAM-BSD-HA were kind gift from C. Doerig (Monash University, Melbourne, Australia) and plasmid pARL was kind gift from the C. Sanchez (Heidelberg, Germany). Protein phosphatase 2A C subunit (human recombinant) was purchased from Cayman Chemical (Ann Arbor, MI 48108, USA). Monoclonal antibodies anti-PP2A alpha, anti-GFP, anti-penta His and horseradish peroxidase-labeled anti-mouse IgG were purchased from Abcam (Cambridge, UK), Roche (Basel, Switzerland), Qiagen (Venlo, The Netherlands) and Santa Cruz Biotechnology (Santa Cruz, CA, USA) respectively.

### 3.2. Phylogenetic Analyses and Secondary Structure Prediction

Protein sequences (listed in Supplemental Table S2) were aligned using the ClustalW algorithm implemented in the BioEdit v7.1 software (Ibis Biosciences, Carlsbad, CA, USA), and manually corrected. Maximum likelihood trees were built using MEGA5 [45] under the JTT + I + G model, with 100 bootstrap repetitions of the following species: *Plasmodium falciparum*, *Plasmodium berghei*, *Plasmodium chabaudi*, *Plasmodium vivax*, *Plasmodium yoelii yoelii*, *Toxoplasma gondii*, *Arabidopsis thaliana*, *Homo sapiens*, *Mus musculus*, *Trypanosoma brucei*, *Tetrahymena thermophila*, *Xenopus laevis*, *Danio rerio*, *Saccharomyces cerevisiae*, *Theileria parva*, *Drosophila melanogaster*, *Leishmania major*, *Oryza sativa*, *Caenorhabditis elegans* and *Schistosoma mansoni*.

PfPTPA secondary prediction was carried out using the ModWeb server [46].

### 3.3. Preparation of Parasites

*P. falciparum* 3D7 clone was grown according to Trager *et al*. [62], in RPMI-1640 medium supplemented with 0.5% AlbuMAXTMII (Invitrogen, Paisley, Scotland, UK), 0.2 mM Hypoxanthin (CCPro, Oberdorla, Germany) and 20 μg/mL Gentamycin (Invitrogen), in the presence of O+ erythrocytes. Cultures were maintained at 37 °C in a humidified athmosphere (5% CO_2_). Parasites were synchronized by a double sorbitol treatment as previously described [63]. To isolate total DNA or protein, parasitized erythrocytes were lysed by saponine [64] and pelleted. Soluble protein extracts were prepared by resuspending parasite pellet in lysis buffer (20 mM Tris-HCl pH 7.4, 150 mM NaCl, 1% Triton X-100 and EDTA-free protease inhibitor cocktail (Roche, Boulogne Billancourt, France) for one hour on wheel at 4 °C followed by a sonication step. Total DNA was extracted using the KAPA Express Extract kit (KAPABioSystem, Montrouge, France) according to the manufacturer’s protocol. Genomic DNA (gDNA) was resuspended parasite pellet in lysis buffer (50 mM Tris-HCl pH 7.5, 150 μg/mL proteinase K and 2% SDS) during 2 h at 55 °C followed by two extraction steps with phenol-chloroform (addition of 1 volume). gDNA was then precipitated with 1/10th volume of sodium acetate and 2 volumes of Ethanol 100%. gDNA was washed twice and resuspended in H_2_O.

### 3.4. Localization of PfPTPA and PfPP2A

All primers used throughout this study are listed in Supplemental Table S1. For an episomal expression of PfPTPA-GFP or PfPP2A-GFP, the full-length coding region of *pfptpa* (Pf3D7_1430100) and *pfpp2a* (Pf3D7_0314400) were amplified from first strand cDNA by PCR using the primers p3-p4 and p25-p26 respectively containing XhoI and KpnI restriction sites. PCR fragments were cloned into PCR2.1-TOPO vector (Invitrogen, San Diego, CA, USA) and their nucleotide sequences were verified by sequencing (Eurofins, Ebersberg, Germany). PCR products were then subcloned in frame with GFP into pARL vector [51] digested with XhoI and KpnI. The plasmid carries the human *dhfr* gene for selection with WR99210 and the *pfcrt* promoter. Ring stages 3D7 parasites were transfected with 100 μg of plasmid DNA by electroporation, according to Sidhu *et al*. [65]. The populations of stably transfected parasites were obtained after six weeks. Live parasites were analyzed and images were recorded by fluorescence microscopy (LSM710, Zeiss, Marly le Roi, France).

### 3.5. Generation of *P. falciparum* Transgenic Parasites

The disruption of *pfptpa* and *pfpp2a* was performed by the insertion of a PCR product corresponding to a 5′ portion from the PfPTPA (765 bp) and PfPP2A (800 bp) sequences into the pCAM-BSD vector that contains a cassette conferring resistance to blasticidin. The insert was obtained using 3D7 genomic DNA as template and the oligonucleotides p5-p6 and p27-p28 which contain PstI and BamHI restriction sites respectively.

*P. falciparum* parasites (ring stages) were transfected as described above. 48 h after transfection, in order to select transformed parasites, blasticidin (Invivogen, San Diego, CA, USA) was added to a final concentration of 2.5 μg/mL. Resistant parasites appeared after three to four weeks and were maintained under drug selection. Populations of stably transfected parasites were obtained after six weeks.

### 3.6. Genotype and Phenotype Analysis of *P. falciparum* Transfectants

Genotype of *pfptpa* and *pfpp2a* knock-out parasites was analyzed by PCR on genomic DNA using the primers p9 and p31 respectively (derived from the 5′ non-translated region and absent in the construct) and p11 specific for the pCAM-BSD vector.

In order to study the phenotype of knock-out parasites, cloning by limiting dilution was performed. Limiting dilutions were set up in 96-well plates at 3% hematocrit in RPMI-1640 medium supplemented with 0.5% AlbuMAXTMII, 0.2 mM Hypoxanthin, 20 μg/mL Gentamycin and 2.5 μg/mL blasticidin. Each well contained 200 μL of medium and an average of 0.1–0.5 parasite. Medium was changed at two or three day intervals beginning at day seven. At day 21, smears were performed to select positive wells. Six positive wells were selected and amplified. Cloning was verified by PCR on genomic extracted DNA with primers p9-p8 for *pfptpa* and p31-p30 for *pfpp2a*.

### 3.7. Recombinant Proteins Expression and Purification

In order to obtain the coding region of PfPTPA, primers p1 and p2 (Supplemental Table S1) were designed according to the sequence of putative PfPTPA (Pf3D7_1430100) available in *Plasmodium* Data Base (PlasmoDB) (http://plasmodb.org/plasmo/). PCR was performed on first strand cDNA from unsynchronized blood cultures of *P. falciparum* 3D7 with the p1 and p2 primers using the Advantage 2 PCR kit (Clontech, St. Germain-en-Layes, France). PCR products were cloned in PCR 2.1-TOPO vector (Invitrogen) and sequenced. Comparative analyses were performed with BIOEDIT software and ClustalW algorithm. After sequence analysis, the coding region of PfPTPA was subcloned in pETDuet-1 which allows the expression of a protein fused with six histidines tag.

With the purpose to produce a recombinant PfPP2A protein, PCR was performed on first strand cDNA from unsynchronized blood cultures of *P. falciparum* 3D7 with the p23 and p24 primers. PCR products were sequenced and subcloned in pETDuet-1 as describe above.

To obtain the PfPTPA mutant constructs, we carried out a PCR-based site-directed mutagenesis strategy using ISIS Proofreading DNA Polymerase. pETDuet-PfPTPA was used as template with primers p13-p14 (PfPTPA E^272^A), p15-p16 (PfPTPA V^283^A), p17-p18 (PfPTPA G^292^A), p19-p20 (PfPTPA M^296^A) and p21-p22 (PfPTPA K^304^A). The PCR conditions consisted of 1 min at 95 °C followed by 16 cycles at 95 °C (30 s), 50 °C (1 min) and 72 °C (9 min). The parental DNA plasmid was digested with DpnI and an aliquot was used to transform XL10-Gold Ultracompetent cells (Stratagene, Amsterdam Zuidoost, The Netherlands). Mutated plasmids were checked by sequencing and then used for the expression of mutated PfPTPA recombinant proteins. A *pfptpa-5mut* gene containing all five mutations with optimized codons has been synthetized (Genscript, Piscataway, NJ, USA) (Sequence presented in Supplemental Figure S3). In order to express PfPTPA-5mut recombinant protein, this synthetic gene was subcloned in pETDuet vector.

Expression of PfPTPA-6His and mutated PfPTPA-6His was carried out in the *E. coli* BL21 strain in the presence of 0.5 mM IPTG at 16 °C overnight. Proteins were extracted in lysis buffer (20 mM Tris-HCl pH 7.4, 150 mM NaCl, 1% Triton X-100, lysozyme 1 mg/mL and Protease inhibitor Cocktail (Roche)) during 1 h at 4 °C on wheel followed by a sonication step. The extract was loaded on a 1 mL nickel Ni-NTA column (Protino NiNTA, Macherey Nagel, Düren, Germany). Washing steps were carried out with a buffer containing 20 mM Tris-HCl pH 7.4, 150 mM NaCl and 20 mM Imidazole. Proteins eluted with Imidazole gradient were dialyzed against 20 mM Tris-HCl pH 7.4, 150 mM NaCl. Under these conditions, the purity checked by SDS-PAGE followed by SimplyBlue™ safe staining (Invitrogen, San Diego, CA, USA) was >90%.

Several attempts to produce PfPP2A were made using pETDuet plasmid containing the coding region of PfPP2A. Unfortunately, we could not produce a concentrated, soluble and active recombinant PfPP2A protein. Considering sequence homology between PfPP2A and mammalian PP2A, activity and interaction tests were performed using *Xenopus* (endogene) or Human PP2A (Cayman, Tallinn, Estonia).

### 3.8. RNA Synthesis of PfPP2A

Capped mRNA (cRNA) was synthetized using a T7 mMessage mMachine kit (Ambion, Austin, TX, USA). cRNA was transcribed from 1 μg of PfPP2A-pGBKT7 linearized by BamHI (Fermentas, St. Leon-Rot, Germany). cRNA was precipitated by 2.5 M LiCl, washed in 70% ethanol resuspended in 20 μL diethyl pyrocarbonate-treated water and quantified by spectrophotometry. Finally 1 μg of cRNA was analyzed with a denaturing agarose gel. Gel staining with 10 μg/mL ethidium bromide allowed confirmation of the size of PfPP2A cRNA and the absence of abortive transcript.

### 3.9. Induction of *Xenopus* Oocytes Germinal Vesicle Breakdown and Co-Immunoprecipitation

Preparation of *Xenopus laevis* oocytes and microinjection experiments were performed as previously described [66]. Two approaches were used.

Briefly, in each assay, 20 oocytes removed from at least two or three animals were pre-injected with His6-PfPTPA (wild type or mutated) recombinant proteins 1 h before incubation with 10 μM of human progesterone (Sigma, St. Louis, MO, USA). Progesterone alone was used as positive control of oocyte maturation. Preliminary experiments using different concentrations ranging from 2 to 40 ng per injection showed that 10 ng of PfPTPA was sufficient to inhibit dramatically progesterone induced GVBD. GVBD was detected by the appearance of a white spot at the apex of the animal pole after 15 h.

In order to carry out immunoprecipitation, oocyte extracts from 20 oocytes removed from at least two or three animals were prepared 15 min after the microinjection of PfPTPA (WT or mutated) as follows: oocytes were lysed in buffer (50 mM HEPES pH 7.4, 500 mM NaCl, 0.05% SDS, 0.5% Triton X100, 5 mM MgCl_2_, 1 mg/mL bovine serum albumin, 10 μg/mL leupeptin, 10 μg/mL aprotinin, 10 μg/mL soybean trypsin inhibitor, 10 μg/mL benzamidine, 1 mM PMSF, 1 mM sodium vanadate) and centrifuged at 4 °C for 15 min at 10,000*g*. To detect His6-PfPTPA proteins, electrophoresis followed by western-blot analysis was performed on oocyte extracts. The membranes were developed with anti-penta His antibodies (Qiagen, Courtaboeuf, France).

A second approach was used in order to test for PfPTPA interaction with PfPP2A. In each assay, 20 oocytes removed from at least two or three different animals were pre-injected with PfPP2A cRNA 18 h before microinjection with wild-type or mutated PfPTPA proteins. As a control, PfPTPA recombinant protein was injected alone. Oocyte extracts were prepared 15 min after microinjection to detect interaction of PfPP2A with PfPTPA proteins. 180 μL of oocyte lysate in PY buffer [67] were incubated with 2 μL of antibodies during 3 h at 4 °C with rotation. Then, 50 μL of 50% solution protein A sepharose (Sigma, St. Louis, MO, USA) was added during 1 h. After three washes of 15 min, beads were resuspended in 6 μL of buffer and electrophoresis followed by western-blot analysis was performed. Co-immunoprecipitations were done using anti-His antibodies (Qiagen, Courtaboeuf, France), anti-Myc (Santa-Cruz Biotechnology, Heidelberg, Germany) or anti-mouse antibodies as control. The membranes were revealed either by anti-Myc antibodies (Santa-Cruz Biotechnology) or by anti-His antibodies (Qiagen) (dilution 1/1000). Antibodies complexes were detected using the Western blotting Luminol Reagent (sc-2048, Santa-Cruz Biotechnology).

### 3.10. Assays for PP2A and Effect of PfPTPA

Phosphatase assays were carried out in buffer containing 40 mM Tris-HCl pH 8.4, 34 mM MgCl_2_, 4 mM EDTA, 2 mM DTT, 0.05 mg/mL Bovine serum albumin (BSA) and 1 mM ATP at 37 °C during 30 min with 20 mM para-NitroPhenyl Phosphate substrat (pNPP). Initial experiments were performed to determine the optimal conditions for human PP2A activity. The reaction was started by adding 20 mM pNPP in a final volume of 200 μL. After an incubation period of 30 min at 37 °C, the liberated para-Nitrophenol was quantified by measurement of absorbance at 405 nm. To investigate the role of PfPTPA on PP2A, 0.04 μg of human PP2A was preincubated with different quantities ranging from 0 to 0.4 μg of recombinant WT or mutated PfPTPA during 30 min at 37 °C before testing the PP2A phosphatase activity.

### 3.11. Measurement of Binding of PfPTPA to PP2A

Binding of PfPTPA to human PP2A was assessed by an ELISA-based assay. Plates were coated with 1 μg/mL of human PP2A or BSA in PBS overnight at 4 °C. Following washings with PBS-Tween 20 0.1%, plates were blocked with PBS-gelatin 1% for 1 h at room temperature. Coated plates where then incubated with different concentrations of PfPTPA (WT or mutated), previously labeled with biotin-*N*-hydroxysuccinimide according to the manufacturer’s instructions (Calbiochem, Nottingham, UK). Incubation of biotinylated-PfPTPA with the different proteins was performed in PBS-Tween 20 0.1% at 37 °C for 2 h. Following five washes (as described above), binding was detected using streptavidin-horseradish peroxidase. After an incubation time of 20 min and five washes, tetramethylbenzidine (TMB) (Uptima, Montluçon, France) was added, and the reaction was stopped by using 2N HCl. The optical density was measured on an ELISA plate reader at 450 nm.

## 4. Conclusions

In this work, we report the first characterization of PfPTPA, the homolog of the human Phosphotyrosyl Phosphatase Activator, a regulator of PP2A. We showed the direct interaction of PfPTPA with PfPP2A and assessed the importance of the V^283^, G^292^ and M^296^ residues in this interaction in absence of another interacting site. As expected, PfPTPA activates PP2A *in vitro* with a critical contribution of the G^292^ residue for this activity. Using the *Xenopus* oocyte model, we observed that the microinjection of PfPTPA blocked the G2/M transition. PfPTPA seems to be essential for the completion of the intraerythrocytic lifecycle of the parasite. However, in order to elucidate the exact role of PTPA in the parasite cell cycle, assays for conditional KO in *Plasmodium berghei* are under investigation. The confirmation of an essential role of PTPA, would suggest the consideration of this protein as a new lead for drug design.
